# The effectiveness of adjuvant radiotherapy after thymoma resection: a systematic review and meta-analysis

**DOI:** 10.1186/s12957-025-04127-z

**Published:** 2025-12-10

**Authors:** Changhao Que, WenLong Chen, Xu Tang, KeYong Li, Wen Sheng Zhou, Qi Deng, Dacheng Jin, ChengFeng Wang, Yunjiu Gou

**Affiliations:** 1 The First Clinical College of Gansu University of Chinese Medicine, Lanzhou, Gansu Province 730000 China; 2 Gansu Chest Disease Clinical Medical Center of Gansu ProvincialHospital, Lanzhou, Gansu Province 730000 China

**Keywords:** Thymoma, PORT, Thymectomy, Systematic review/Meta-analysis

## Abstract

**Introduction:**

Our systematic review and meta-analysis aimed to investigate the impact of postoperative radiotherapy(PORT) on patient outcomes following thymoma resection.

**Methods:**

A comprehensive search of PubMed, EMbase, The Cochrane Library, Web of Science, Wanfang, Wipo, and China Knowledge Network databases was systematically conducted. Relevant literature comparing the therapeutic efficacy and effectiveness of thymectomy and thymectomy+ PORT in patients with thymoma published until August 2024 was gathered. The retrospective studies included in this analysis were evaluated using the Newcastle-Ottawa Scale (NOS), while meta-analysis of the literature was performed using Review Manager 5.4 software.

**Results:**

A total of 23 retrospective studies, encompassing 13,742 patients, were included in the analysis. Among them, 6,980 patients belonged to the surgery-only group (3,321 male patients and 3,659 female patients), with an average age of 54.08 years. The surgery + PORT group consisted of 6,762 patients (3,385 male patients and 3,377 female patients) with an average age of 53.76 years. All included studies had a NOS score of ≥7.The meta-analysis results revealed that, in comparison to the surgery group, the surgery + PORT group exhibited higher rates of overall survival at 1 year, 3 years, 5 years, and 10 years. Additionally, the surgery + PORT group demonstrated superior disease-free survival (DFS)rates at 1 year and 5 years as well as disease-specific survival (DSS) rates at 3 years, 5 years, and 10 years. However, no significant differences were observed between the two groups in terms of DFS at 3 years or DSS at 1 year.

**Conclusions:**

Available retrospective evidence indicates an association between PORT and improved survival after thymoma resection, though prospective validation is warranted.

**Supplementary Information:**

The online version contains supplementary material available at 10.1186/s12957-025-04127-z.

## Introduction

 Thymomas, originating from thymic epithelial cells, are the most common tumors of the anterior mediastinum despite their overall rarity [1]. Thymoma is rare, with an incidence of approximately 2–4 per million population per year, and typically affects individuals aged 40–70 years without significant gender differences [2]. Complete surgical resection remains the gold standard for cure across all stages. Chemotherapy is commonly used as neoadjuvant therapy before surgery or as definitive treatment for unresectable tumors [3].While the role of radiotherapy in thymoma management is established, sufficient large randomized clinical trials are lacking to confirm whether PORT improves survival after thymoma resection—making this topic a subject of ongoing debate. Due to the complexity of mediastinal anatomy, complete resection is not always feasible; rare cases of local recurrence also occur, which are often incurable. Thus, PORT may be considered to enhance local control, though caution is needed regarding potential toxicity, given the proximity of critical structures and prolonged clinical trajectories [4].In recent years, several studies [5, 6] have explored PORT’s potential benefits in thymoma patients. However, most of these were retrospective single-center analyses with small sample sizes, yielding inconsistent results. Consequently, whether adjuvant PORT confers survival benefits—including improvements in Overall Survival (OS), DSS, and DFS—across different thymoma stages remains controversial.

This study incorporates the most up-to-date literature to conduct a meta-analysis on the efficacy of PORT in thymoma patients across various Masaoka stages, aiming to provide substantiated evidence for its clinical application.

## Materials and methods

### Literature inclusion and exclusion criteria

Patient grouping: Group A included thymoma patients who underwent surgery only; Group B included thymoma patients who received both surgery and PORT.

Inclusion criteria: (1) The included studies must be retrospective, prospective, or randomized controlled trials. (2) Study subjects will include (A) patients diagnosed with thymoma who have undergone thymectomy, and (B) Patients diagnosed with thymoma who have undergone thymectomy and PORT. Outcome metrics will include at least one of the following: 1, 3, 5, or 10 year OS, DFS, and DSS.

Criteria for exclusion: (1) case reports, systematic reviews, and meta-analyses, among other types of literature; (2) non-English language literature; (3) interventions involving preoperative chemotherapy or radiotherapy; (4) literature with an NOS score below 7; (5) data that could not be extracted or original text that was inaccessible; (6) Studies including patients with thymic carcinoma were excluded.(7)duplicate publications.

### Literature search methodology

We conducted a comprehensive search using a combination of controlled vocabulary and free-text terms in PubMed, EMbase, The Cochrane Library, Web of Science, and Wanfang, Wipu, and China Knowledge databases to retrieve publicly available literature that compared the efficacy and effectiveness of thymectomy versus thymectomy+PORT in patients diagnosed with thymoma from inception until August 2024. The primary search terms utilized in English were: Thymoma, PORT, and Radiotherapy.The detailed search strategy is provided in the accompanying search strategy table.

### Literature screening and data extraction

Two thoracic surgeons independently reviewed the literature and extracted data, cross-referencing for any discrepancies. In the event of disagreement, a third surgeon was invited to participate in the discussion and decision-making process. The literature was analyzed for fundamental information including the primary author, study design type, country of origin, year of publication, duration of follow-up, and tumor size. Additionally, the number of patients receiving different interventions, male-to-female ratio, presence of comorbid myasthenia gravis (MG), and Masaoka staging were recorded. Outcome indicators extracted from the literature encompassed 1, 3, 5, and 10 year OS, DFS, and DSS.

### Quality evaluation of included studies

The quality of the retrospective studies included in the analysis was assessed using the NOS. Two thoracic surgeons independently evaluated the literature for its quality, while a third thoracic surgeon was invited to participate in discussions and decision-making in cases of disagreement.

### Statistical analyses

A meta-analysis was conducted using Review Manager 5.4 software, supported by the Cochrane Collaboration, to analyze the included data. The effect indicators assessed were exclusively dichotomous data, with odds ratios (OR) and their corresponding 95% confidence intervals (CI) calculated. The survival outcomes in this study were pooled using OR, primarily because some of the included studies did not report hazard ratios (HRs) or provide survival curve data from which HRs could be estimated. OR capture the strength of association with survival events at a specific time point but do not account for the timing or temporal dynamics of event occurrence. Therefore, the interpretation of OR should be approached with caution.Following the analysis, a heterogeneity test was performed to assess whether there was significant variation among the studies, indicated by an I² value less than 50% and P value greater than 0.1. In case of no significant heterogeneity, a fixed-effects model was employed for data analysis. If substantial heterogeneity between studies existed (I² ≥ 50% and *P* ≤ 0.1), a random effects model was utilized for data analysis instead. Sensitivity analysis was conducted to evaluate result stability while publication bias detection relied on funnel plot method.

### Protocol registration number and link

PROSPERO 2025 CRD420251138474.Available from https://www.crd.york.ac.uk/PROSPERO/view/CRD420251138474.

## Results

### Literature search of results

The search strategy, developed prior to the study, served as a reference and yielded 651 documents. After removing duplicates, 464 documents remained. Further exclusion of documents based on title and abstract resulted in a final selection of 104 relevant articles. Finally, applying the pre-established inclusion criteria to the full text allowed us to analyze a total of 23 selected publications [5–27]. The literature included in this analysis consisted solely of retrospective studies. Given that all the included studies were retrospective in nature, we performed an observational meta-analysis.The screening process is presented in the accompanying table titled “Screening Process.”

### Quality assessment of the included literature

The retrospective studies included in this analysis all achieved a NOS score of ≥7, indicating the inclusion of high-quality literature. Please refer to Table 1 for basic information on the included studies and consult Table 2 for their respective scores.

**Table 1:** Basic information on the included literature

First Author	nation	Number of patients(group A/B)	gender(M/F)	age(group A/B)	follow-up time(month)	Masaoka Stage(group A/B)				extent of resection			histology subtype						radiotherapy dose
						Ⅰ	Ⅱ	Ⅲ	Ⅳ	R0	R1	R2	A	AB	B1	B2	B3	NOS	
Liao 2018 [5]	China	25/74	A:11/14 B:39/35	49/52	3–155	NA				99	0	0	12		51		36	0	50-56GY
Zhou 2022 [6]	China	122/83	A:56/66 B:41/42	51/57	84.3(6–279)	0/0	93/49	29/34	0/0	205	0	0	113			92		0	39.2–63.4 Gy
Chang 2011 [7]	Korea	17/59	A:11/6 B:29/30	45/52	58.5(6–231)	0/0	15/50	2/9	0/0	76	0	0	48			28		0	43.2–66 Gy
Chen 2023 [8]	China	54/72	A:20/34 B:35/37	46.6±10.0/44.2±10.7	23(1–139)	23/9	12/29	11/26	5/3	126	0	0	9	16	17	60	24	0	50–54 Gy
Chen 2010 [9]	China	41/66	A:24/17 B:30/36	44/49	63(2–303)	0/0	41/66	0/0	0/0	107	0	0	10	48	26	12	11	0	22–60 Gy
Fan 2013 [10]	China	12/53	A:7/5 B:26/27	53/46	50(5–360)	0/0	0/0	12/53	0/0	65	0	0	2	7	12	28	16	0	28–60 Gy
Fernand 2010 [11]	USA	346/669	NA	60/55	65(1–361)	NA													
Jackson 2017 [12]	USA	1587/1444	A:729/828 B:706/738	NA	57.2(1–129)	1087/790		317/451		2463	476	92	NA						
Kondo 2003 [13]	Japan	35/105	NA	53.7±14.0	NA					140	0	0	15	42	29	41	13	0	36–53.8 Gy
Lim 2015 [14]	Korea	184/184	A:97/87 B:97/87	57	52(6–211)	NA							40	87	67	60	114	0	NA
Lin 2023 [15]	China	262/262	A:123/139 B:127/135	51.7±10.2/53.3±11.7	NA								51	115	94	108	156	0	NA
Lu 2018 [16]	China	24/150	A:9/15 B:70/80	NA	15–144	9/27	13/81	2/42	0/0	174	0	0	1	32	46	58	37	0	45–55 Gy
Mou 2021 [17]	China	1125/1111	A:544/581 B:539/572	NA	50(3–221)	NA		121/109		NA									
Omasa 2015 [18]	Japan	787/323	A:333/454 B:144/179	58.4±13.5/54.0±12.7	NA	0/0	640/200	147/123	0/0	1088	22	0	72	298	249	313	178	0	NA
Rena 2007 [19]	Italy	32/26	A:17/15 B:13/13	52.1±10.9/50.2±15.6	91(9–171)	0/0	32/26	0/0	0/0	58	0	0	3	23	19	12	1	0	45–54 Gy
Rimner 2016 [20]	USA	574/689	A:253/321 B:381/308	59/51	48	0/0	449/421	125/268	0/0	1263	0	0	360		827			76	NA
Singha 2003 [21]	USA	47/23	A:15/32 B:16/7	59.4	70.3	27/3	20/20	0/0	0/0	70	0	0	12	9	27	17	5	0	45–54 Gy
Song 2020 [22]	Korea	202/202	A:97/105 B:97/105	51.5/50	58.2(2–211)	0/0	174/172	28/30	0/0	404	0	0	236			168		0	NA
Wen 2018 [23]	China	119/75	A:71/48 B:49/26	NA	38(1–174)	NA		37/47		NA									
Yan 2016 [24]	USA	57/31	NA	57	42.8(1–198)	31/2	13/9	5/13	8/7	88	0	0	60			28		0	45–70 Gy
Yang 2023 [25]	China	455/182	A:258/197 B:101/81	60	39	NA													
Yuan 2018 [26]	China	90/96	A:48/42 B:48/48	50.9±12.0	240	NA													
Zhang 2022 [27]	China	783/783	A:424/359 B:415/368	59.7±14.1	66.7(2–168)	637/629		126/139		1465	72	29	166	462	239	374	325	0	NA

Group A: simple surgery group; Group B: Surgery + PORT group; MG: myasthenia gravis; PORT: Postoperative adjuvant radiation therapy

**Table 2:** The NOS score of the included literature

First Author	Selection of study populations				Comparability	Measurement of exposure factors				Score
	Enrollment diagnosis	Representation	Control selection	Contrast definition		Proven track record/Blinded structuring		Methods of exposure	No response rate	
Yuan 2018 [26]	0	1	1	1	0	1	1	1	1	7
Zhou 2022 [6]	0	1	1	1	1	1	1	1	0	7
Yang 2023 [25]	1	1	1	1	1	0	1	1	0	7
Zhang 2022 [27]	1	1	1	1	1	0	1	1	1	8
Liao 2018 [5]	0	1	1	1	0	1	1	1	1	7
Song 2020 [22]	1	1	1	1	1	0	1	1	0	7
Lu 2018 [16]	1	1	1	1	1	0	1	1	0	7
Chen 2023 [8]	1	1	1	1	1	1	1	1	0	8
Mou 2021 [17]	1	1	1	1	1	1	1	1	0	8
Wen 2018 [23]	1	1	1	1	0	1	1	1	0	7
Lin 2023 [15]	1	1	1	1	0	1	1	1	0	7
Rimner 2016 [20]	1	1	1	1	1	0	1	1	0	7
Lim 2015 [14]	0	1	1	1	1	1	1	1	1	8
Fan 2013 [10]	1	1	1	1	0	1	1	1	0	7
Omasa 2015 [18]	1	1	1	1	0	1	1	1	0	7
Chang 2011 [7]	0	1	1	1	0	1	1	1	1	7
Kondo, K. 2003 [13]	1	1	1	1	0	1	1	1	0	7
Fernandes, A. T.2010 [11]	0	1	1	1	1	1	1	1	1	8
Chen, Y. D.2010 [9]	0	1	1	1	0	1	1	1	1	7
Jackson, M. W. 2017 [12]	0	1	1	1	1	1	1	1	0	7
Rena, O. 2007 [19]	1	1	1	1	1	0	1	1	0	7
Singhal, S.2003 [21]	0	1	1	1	1	0	1	1	1	7
Yan, J. 2016 [24]	1	1	1	1	1	0	1	1	1	8

### Meta-analysis results

**Fig. 1:**
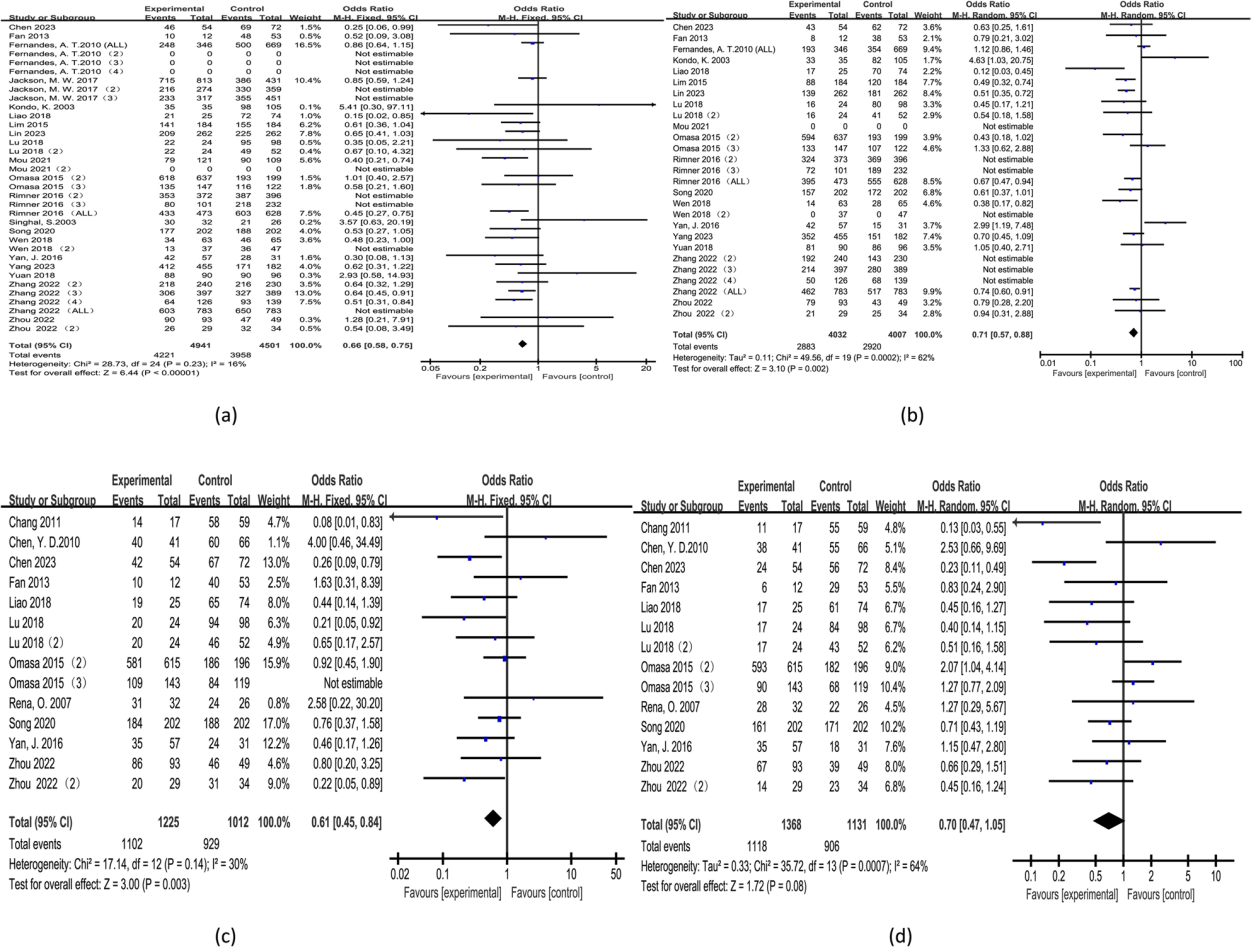
The forest plot of the meta-analysis. **a**: 5 year OS; **b**: 10 year OS; **c**: 5 year DFS; **d**: 10 year DFS

We conducted a comprehensive meta-analysis of OS, DFS, and DSS among the included studies. Of the 23 studies analyzed, 20 reported OS data [5, 6, 8, 10–18, 20–27], 11 reported DFS data [5–10, 16, 18, 19, 22, 24], and 9 reported DSS data [5, 9, 10, 14, 15, 17, 23, 25, 27]. The OS analysis encompassed 10,972 patients, the DFS analysis included 2,499 patients, and the DSS analysis covered 4,620 patients. However, due to the extended 10 year follow-up period, the number of patients contributing to the 10 year estimates for these outcomes was slightly lower compared to other time points. Detailed findings are summarized in the Table 3.The forest plot of the meta-analysis is shown in Figure 1.All forest plots are available in the supplementary materials.

**Table 3:** Results of meta-analysis of outcome measures

Outcome measures	Number of documents	Number of people in each group(example)		heterogeneity		Effect model	OR	95%CI	*P*	Whether PORT is Supported
		Surgery	Surgery+PORT	I²(%)	*P*					
OS One-year	20 [5, 6, 8, 10–18, 20–27]	5 589	5 383	3	0.420	fixed-effect	0.32	(0.25,0.42)	<0.001	YES
Three-year	20 [5, 6, 8, 10–18, 20–27]	5 589	5 383	0	0.720	fixed-effect	0.55	(0.48,0.64)	<0.001	YES
Five-year	20 [5, 6, 8, 10–18, 20–27]	4 941	4 501	16	0.230	fixed-effect	0.66	(0.58,0.75)	<0.001	YES
Ten-year	17 [5, 6, 8, 10, 11, 13–16, 18, 22–27]	4 032	4 007	62	<0.001	random	0.71	(0.57,0.88)	0.002	YES
DFS One-year	11 [5–10, 16, 18, 19, 22, 24]	1 368	1 131	0	0.710	fixed-effect	0.47	(0.23,0.93)	0.030	YES
Three-year	11 [5–10, 16, 18, 19, 22, 24]	1 368	1 131	0	0.560	fixed-effect	0.86	(0.61,1.22)	0.400	NO
Five-year	11 [5–10, 16, 18, 19, 22, 24]	1 225	1 012	30	0.140	fixed-effect	0.61	(0.45,0.84)	0.003	YES
Ten-year	11 [5–10, 16, 18, 19, 22, 24]	1 368	1 131	64	<0.001	random	0.70	(0.47,1.05)	0.080	NO
DSS One-year	9 [5, 9, 10, 14, 15, 17, 23, 25, 27]	2 316	2 304	0	0.580	fixed-effect	0.83	(0.55,1.26)	0.380	NO
Three-year	9 [5, 9, 10, 14, 15, 17, 23, 25, 27]	2 316	2 304	0	0.820	fixed-effect	0.44	(0.35,0.55)	<0.001	YES
Five-year	9 [5, 9, 10, 14, 15, 17, 23, 25, 27]	2 316	2 304	19	0.260	fixed-effect	0.53	(0.44,0.63)	<0.001	YES
Ten-year	9 [5, 9, 10, 14, 15, 17, 23, 25, 27]	2 091	2 070	76	<0.001	random	0.53	(0.35,0.82)	0.004	YES

*PORT* Postoperative radiation therapy, *DSS* Disease-specific survival rates, *OS* Overall survival rate, *DFS* Disease-free survival

Among the 23 included studies, 8 discussed various surgical approaches and methods, including median sternotomy, transcervical thymectomy, thoracotomy, and video-assisted thoracoscopic surgery [5–8, 16, 19, 21, 25]. Only one study indicated that advanced-stage patients were more likely to undergo more extensive procedures, such as extended thymectomy, whereas early-stage patients predominantly received thymomectomy or thymectomy [25]. Another study reported that differences existed between groups prior to propensity score matching, particularly in the extent of surgical intervention between the PORT and non-PORT groups; these differences were balanced after matching [5]. The remaining studies did not address variations in surgical approaches or the potential impact of different techniques.

Among the 23 included studies, 11 referenced the extent of resection. Only one study reported that extended thymectomy demonstrated significantly better efficacy (96.47% vs. 68.18%, *P* < 0.001)^16^; however, this did not translate into a significant improvement in the oncological outcomes for thymoma. Another study differentiated between complete thymectomy and partial resection, reporting a higher rate of complete resection, but found no significant differences in the extent of resection across groups [17]. The remaining nine studies [7, 9, 13, 19–22, 25, 27] did not clearly specify such distinctions and merely emphasized the term “complete resection.”

### Subgroup analysis

**Fig. 2:**
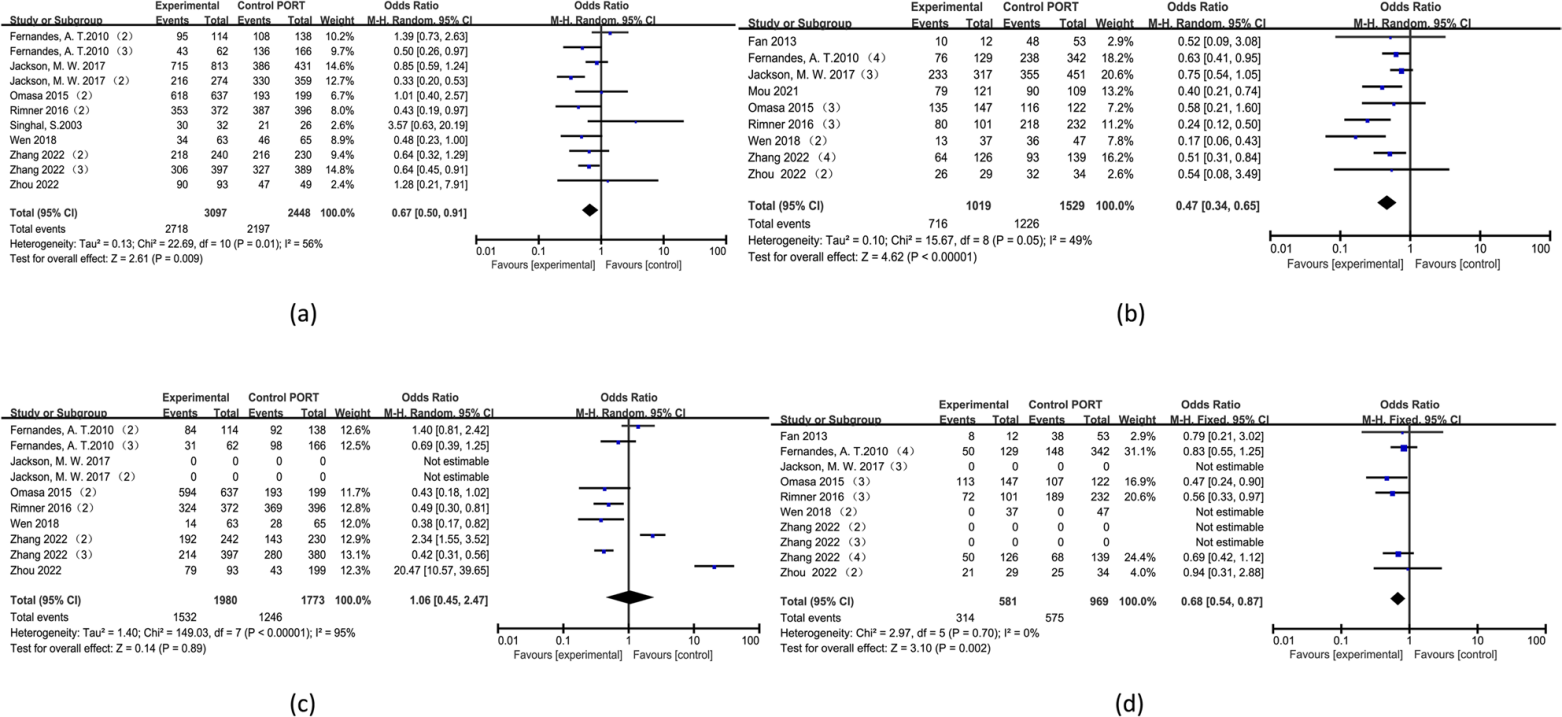
The forest plot of the subgroup analysis. **a**: 5 year OS(Ⅰand Ⅱ); **b**: 5 year OS(Ⅲand Ⅳ); **c**: 10 year OS(Ⅰand Ⅱ); **d**: 10 year OS(Ⅲand Ⅳ).

To mitigate bias in subgroup analysis, we conducted a targeted review of the literature [6, 10–12, 17, 18, 20, 21, 23, 27] focusing on extractable OS data for different Masaoka stages. The findings are presented in Table 4. Patients with stage I and II disease exhibited significantly higher 1, 3, and 5 year OS rates in the surgery + PORT group compared to the surgery-only group (*P* < 0.05). However, there was no statistically significant difference between the two groups regarding 10 year OS rates (*P* >0.05). For patients with stage III and IV disease, the surgery+PORT group demonstrated significantly superior 1 year through 10 year OS rates compared to the surgery-only group (*P* < 0.05). In terms of longitudinal comparison between patients with stages I and II versus those with stages III and IV disease, PORT played a more prominent role in enhancing 1 year OS rates among stages I and II patients; whereas for 3 year through 10 year OS rates PORT had a more pronounced impact among stages III and IV.The forest plot of the subgroup analysis is shown in Figure 2.All forest plots are available in the supplementary materials.

**Table 4:** Stage I, II and stage III, IV results of subgroup analysis of patients

Outcome measures	Number of documents	Number of people in each group(example)		heterogeneity		Effect model	Effect size		95%CI	*P*-value	Whether PORT is Supported
		surgery	Surgery+PORT	I²(%)	*P*-value						
One-year OS											
Stage Ⅰ and Ⅱ	8	3097	2448	1	0.430	fixed-effect	OR=0.23	(0.15,0.37)		<0.001	YES
Stage Ⅲ and Ⅳ	9	1019	1529	16	0.30	fixed-effect	OR=0.37	(0.25,0.55)		<0.001	YES
Three-year OS											
Stage Ⅰ and Ⅱ	8	3097	2448	0	0.650	fixed-effect	OR=0.60	(0.48,0.75)		<0.001	YES
Stage Ⅲ and Ⅳ	9	1019	1529	4	0.40	fixed-effect	OR=0.44	(0.35,0.56)		<0.001	YES
Five-year OS											
Stage Ⅰ and Ⅱ	8	3097	2448	56	0.010	random	OR=0.67	(0.50,0.91)		0.009	YES
Stage Ⅲ and Ⅳ	9	1019	1529	49	0.050	random	OR=0.47	(0.34,0.65)		<0.001	YES
Ten-year OS											
Stage Ⅰ and Ⅱ	6	1980	1773	95	<0.001	random	OR=1.06	(0.45,2.47)		0.890	NO
Stage Ⅲ and Ⅳ	7	581	969	0	0.700	fixed-effect	OR=0.68	(0.54,0.87)		0.002	YES

*PORT* Postoperative radiation therapy, *OS* Overall survival rate

### Sensitivity analysis

**Fig. 3:**
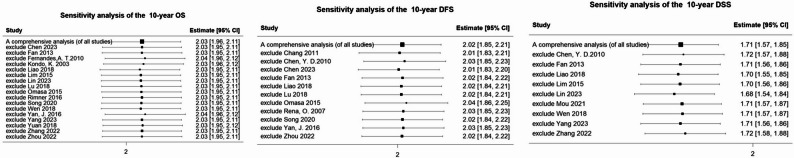
The results of the sensitivity analysis

After systematically eliminating each piece of literature and conducting a comprehensive analysis of the remaining data, no significant disparities were observed among the results. These findings demonstrate the robustness of the conclusions drawn from this meta-analysis. See Figure 3 for details.

### Publication bias

**Fig. 4:**
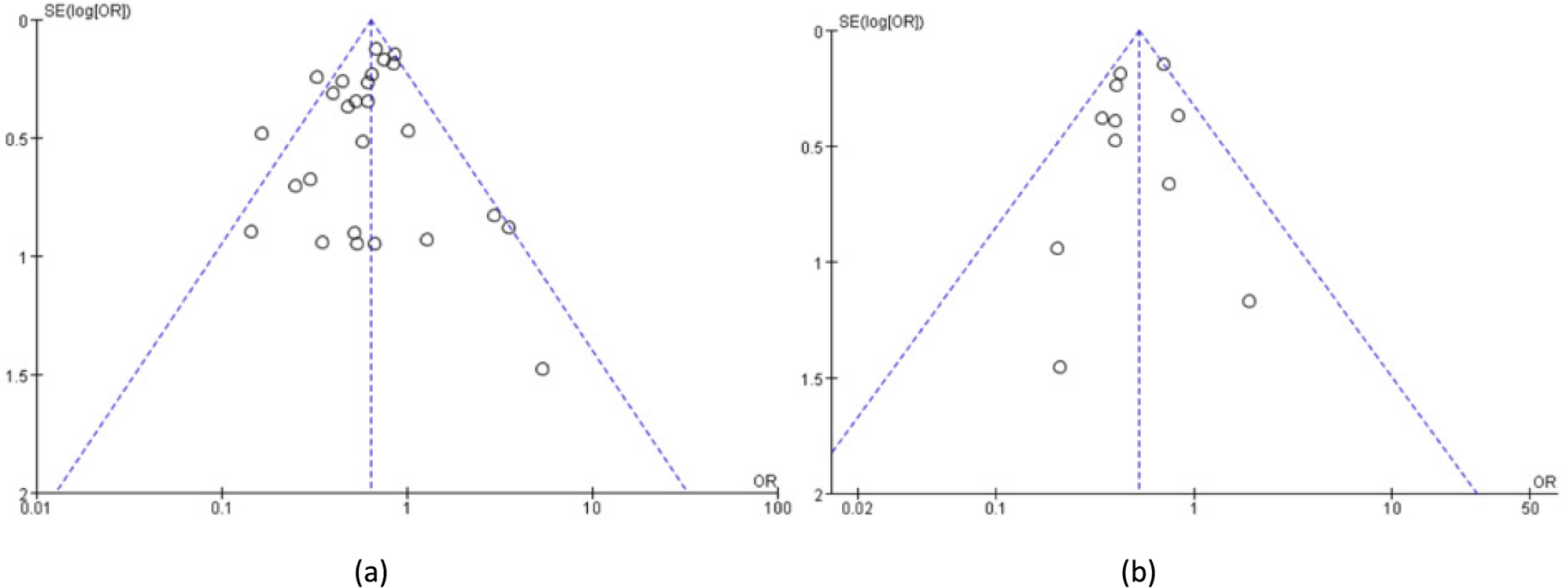
Publication bias analysis.** a**: Funnel plot of 5 year OS rate for all patients;** b**: Funnel plot of 5 year DSS rate for all patients

Publication bias analysis of the included indicators was conducted using funnel plots in Review Manager 5.4, supported by the Cochrane Collaboration network. Taking 5 year OS rate and 5 year DSS rate as examples, the distribution of included documents on both sides of the funnel plot was balanced. The descriptive meta-analysis showed minimal evidence of publication bias; refer to Figure 4.

## Discussions

Thymomas are a relatively uncommon type of solid tumor in the thoracic region. Currently, all subtypes of thymic epithelial tumors (TETs) are considered to possess malignant potential. Even patients diagnosed with type A thymoma may experience distant metastasis, and early-stage TETs that have been completely resected can still recur. Conversely, TETs exhibit a relatively indolent behavior, as patients often survive for extended periods following disease progression or recurrence. This characteristic poses significant challenges in conducting large-scale prospective randomized studies to obtain high-quality evidence and guide clinical practice effectively. Consequently, the diagnosis and treatment of TETs remain contentious, relying primarily on empirical approaches. The current guidelines provided by the National Comprehensive Cancer Network (NCCN) also heavily rely on expert opinions [28].

In a previous study [29], the comparison of OS between patients with stage II-III thymomas who received PORT and those who did not receive it revealed that PORT conferred a significant benefit to OS in patients undergoing complete resection for stage II/III thymoma. However, this analysis included only five studies encompassing a total of 4,746 patients, out of which only two studies provided data on DFS.

The present study included twenty-three high-quality English publications on retrospective studies, encompassing a total of 13,742 patients across all stages (Ⅰ-Ⅳ) of the disease. The outcome measures assessed were OS, DFS, and DSS. This study offers several advantages over similar research: firstly, it incorporates a larger number of up-to-date publications, selecting 23 articles with scores ≥7 based on the NOS [30], with the most recent publication dated April 2023 ^15^. Around 2003, the use of enhanced chest CT in the preoperative staging of thymoma gradually became standardized [31]. After 2010, PET-CT began to be widely applied in the staging of advanced thymoma, particularly in cases suspected of large vessel invasion or distant metastasis [32]. The International Thymic Malignancy Interest Group (ITMIG) also issued preliminary consensus guidelines on the diagnostic staging criteria for CT and PET-CT, which further reduced technical variability across different medical centers [33]. These developments ensured a high degree of consistency in the staging methods used across the included literature, while the 20 years study period helped guarantee an adequate sample size.Lastly, this study expands upon previous work by including twelve outcome measures categorized into three domains and collecting more detailed data on outcomes (Table 3), thereby providing an objective evaluation of the efficacy and survival profile of PORT in thymoma patients.

The 1, 3, 5 and 10 year OS rates in the surgery + PORT group demonstrated over those of the surgery alone group, consistent with findings from two previous meta-analyses [29, 34]. OS is widely regarded as the most reliable efficacy endpoint in tumor clinical trials and preferred when evaluating patient survival comprehensively. Even a slight improvement in OS can be considered as evidence of significant direct clinical benefits. The 5 year OS rate is commonly utilized in clinical practice to assess the proportion of tumor patients who survive for more than five years following comprehensive treatments. Recurrence and metastasis occur frequently within three and five years after radical surgery, accounting for approximately 80% and 10%, respectively. Therefore, statistics on the rates of OS at intervals of one, three, five, and ten years hold considerable clinical value.

The 1 and 5 year DFS rates were superior in the surgery+PORT group compared with the surgery-only group, though no statistically significant differences were observed in 3 and 10 year DFS between the two groups. Three potential explanations for this finding are proposed: first, the sample size was inadequate, with relatively less DFS data than OS data—this may have limited the ability to detect between-group differences; second, insufficient baseline data prevented subgroup analysis of DFS during the initial study; third, the effects of surgery and radiotherapy on DFS may exhibit temporal variations.Given the slow clinical course of thymoma, extending follow-up could better clarify disparities in DFS outcomes, as longer follow-up may capture late recurrence events missed in short-term studies. In our analysis of 10 year DFS, significant heterogeneity was observed across literature sources (I²= 64%). Sensitivity analysis (excluding each source sequentially and re-analyzing the remaining data) failed to identify a clear origin of this heterogeneity, with I²remaining >50% throughout. Notably, one study [24]highlights positive surgical margins as a key factor influencing thymoma patients’DFS; however, this variable was not included in our analysis, and confounding effects related to surgical margin status cannot be ruled out.Collectively, our findings suggest that the potential positive impact of PORT on DFS requires further verification via prospective studies.

The 3, 5 and 10 year DSS rates of the surgery + PORT group were significantly higher than those of the surgery group, while there was no statistically significant difference in the 1 year DSS between the two groups. DSS criteria in this study focused on deaths caused by thymoma recurrence or metastasis. It is important to note that DSS specifically considers deaths related to a particular disease, excluding other causes. Therefore, DSS accurately reflects clinical benefits. However, variations exist in the judgment criteria for DSS. Although PORT did not demonstrate a clear advantage in terms of 1 year DSS due to rare occurrences of thymoma-related deaths within this timeframe regardless of clinical intervention, it should be emphasized that thymoma has a slow clinical course and patients generally have long mean survival rates. Hence, evaluating PORT’s impact on long-term efficacy is crucial for patients with thymoma. OR for 1, 3, 5 and 10 year DSS were calculated as 0.83, 0.44, 0.53, and 0.53 respectively without exhibiting a linear relationship or an evident trend towards increased benefit as survival time prolongs further; these findings warrant further discussion in future studies.

Subgroup analysis demonstrated that PORT showed efficacy in patients with Stage III and Stage IV disease. Several studies conducted by scholars at the University of Pennsylvania [7, 35–38] have demonstrated no local control benefit of PORT in stage II thymoma. Furthermore, other literature [39, 40]has indicated that PORT is not independently associated with disease progression in multivariate analyses involving stage II and III patients. Consequently, there exists a clear controversy regarding the role of PORT in patients with stage II and III. According to our expert consensus on the diagnosis and treatment of thymic epithelial tumors, PORT is not recommended for stage I patients following complete resection (R0). However, thymomas with specific World Health Organization (WHO) subtypes, such as types B2 and B3, tend to exhibit more aggressive biological behavior. Clinicians may therefore perceive these cases as having a higher risk of recurrence and recommend adjuvant radiotherapy. Furthermore, if microinvasion is suspected during surgery or the resection margin is close—despite a final pathological report indicating R0 resection—physicians may still opt for PORT to enhance local control. Consequently, the stage I patients who received PORT in this study likely represent a highly selected subgroup with an inherently higher baseline risk of recurrence, and their prognostic features differ from those of stage I patients who did not undergo radiotherapy [41].The use of PORT for R0-resected stage II-III patients remains controversial, particularly for stage II patients after R0 resection. Radiotherapy is routinely recommended for stage III and IV patients. To validate this conclusion, further clinical randomized trials are warranted. We intend to conduct relevant trials when circumstances permit in order to obtain the most objective and scientifically rigorous clinical evidence.

The present study had limitations. Firstly, the original studies included in this meta-analysis were retrospective studies with inherent selection bias and information bias, and the conclusions reached need to be validated by future randomized controlled trials with larger sample sizes. Secondly, among the general data collected, important variables such as disease stages, histology, completeness of resection, and the presence of MG had varying effects. Notably, only two of the included studies specifically analyzed thymoma patients with concomitant MG [8, 16]. Regarding postoperative care, most studies did not clearly specify the potential impact of variations in postoperative management approaches. As a result, we were unable to collect sufficient data for a comprehensive analysis. Additionally, the lack of certain data in several radiotherapy reports inevitably introduced some degree of bias into our findings. Lastly, only English publications were considered for inclusion in this meta-analysis due to language restrictions; however, publications written in other languages may also meet eligibility criteria but were not included. Despite these limitations mentioned above, the present study provides novel insights into PORT’s efficacy.

Further experimental demonstrations are necessary to verify the efficacy of PORT in patients with thymoma. Due to insufficient data collected on individually staged patients in this study, we grouped stage I and II patients together with stage III and IV patients for subgroup analysis. It would be ideal to analyze the efficacy of PORT separately for Masaoka stage I-IV patients, in order to validate the benefit of PORT for each individual patient and explore the impact of performing PORT at different stages of thymoma. However, due to variations in publication dates, countries, and therapeutic strategies among the included literature, standardizing the details of PORT for thymoma was not feasible in our study. Future studies should focus on exploring specific procedures such as timing, drug dosage, and irradiation duration for PORT in thymoma. This will enable the formulation of more personalized treatment protocols tailored to different patients with thymoma, thereby maximizing the effects of PORT. Given that thymoma is a relatively rare condition with limited cases available for study and its therapeutic effect takes time to confirm due to its slow clinical course, we advocate conducting more prospective studies through multi-center collaborations using national databases across various platforms. These efforts will facilitate verification of therapeutic efficacy at different phases and promote standardization in diagnosis and treatment approaches for thymoma while improving patient survival rates and ensuring their quality of life. Although no large prospective studies with definitive conclusions on the clinical benefits of PORT for patients after thymoma resection have been reported yet, the available retrospective studies suggest a significant role for PORT. It is believed that PORT enhances efficacy in patients following thymoma resection, and further robust evidence is anticipated to support this conclusion. This study aims to contribute valuable insights into the application of PORT in the clinical diagnosis and treatment of thymoma.

## Conclusions

Adjuvant radiotherapy following thymoma resection demonstrates superior outcomes compared to surgery alone, with improved OS, 1 year and 5 year DFS, as well as enhanced 3 year, 5 year, and 10 year DSS.Retrospective evidence to date supports an association between PORT and improved survival outcomes following thymoma resection, while prospective studies are needed to validate this finding.

## Supplementary Information

Supplementary Material 1.

Supplementary Material 2.

Supplementary Material 3.

Supplementary Material 4.

Supplementary Material 5.

Supplementary Material 6.

Supplementary Material 7.

Supplementary Material 8.

Supplementary Material 9.

Supplementary Material 10.

Supplementary Material 11.

Supplementary Material 12.

Supplementary Material 13.

Supplementary Material 14.

Supplementary Material 15.

Supplementary Material 16.

Supplementary Material 17.

Supplementary Material 18.

Supplementary Material 19.

Supplementary Material 20.

Supplementary Material 21.

Supplementary Material 22.

Supplementary Material 23.

Supplementary Material 24.

Supplementary Material 25.

Supplementary Material 26.

Supplementary Material 27.

## Data Availability

Data extracted from included studies, some can be queried from databases such as pubmed. Access to statistics is limited and can be obtained by contacting gouyunjiu@163.com.
