# Supplementary material for: The effectiveness of adjuvant radiotherapy after thymoma resection: a systematic review and meta-analysis
Source: World J Surg Oncol. 2025 Dec 10;24:36. doi: 10.1186/s12957-025-04127-z (PMC12801928; doi:10.1186/s12957-025-04127-z)
Supplement: Supplementary file 1 — Supplementary Material 1. [file 12957_2025_4127_MOESM1_ESM.docx]

Pubmed:

#1 Thymoma[MeSH Terms]

#2 Thymoma[Title/Abstract]

#3 "Carcinoma, Thymic"[MeSH Terms]

#4 "Carcinoma, Thymic"[Title/Abstract]

#5 "Thymic Carcinoma"[Title/Abstract]

#6 #1 OR #2 OR #3 OR #4 OR #5

#7 "Postoperative Radiotherapy"[MeSH Terms]

#8 "Postoperative Radiotherapy"[Title/Abstract]

#9 "Radiotherapy"[MeSH Terms]

#10 "Radiotherapy"[Title/Abstract]

#11 "Radiation Treatment"[Title/Abstract]

#12 "Targeted Radiotherapy"[Title/Abstract]

#13 #7 OR #8 OR #9 OR #10 OR #11 OR #12

#14 "Myasthenia Gravis"[MeSH Terms]

#15 "Myasthenia Gravis"[Title/Abstract]

#16 #14 OR #15

#17 #6 AND #13

#18 #17 AND #16

#19 #17 NOT #16

#20 #18 OR #19

#21 Filters: Humans, English, All article types

EMbase

1. 'thymoma'/exp OR thymoma

2. 'thymic carcinoma'/exp OR 'thymic carcinoma*'

3. 1 OR 2

4. 'postoperative radiotherapy'/exp OR 'postoperative radiotherapy'

5. 'radiotherapy'/exp OR radiotherapy

6. 'radiation therapy'/exp OR 'radiation treatment*'

7. 'targeted radiotherapy'/exp OR 'targeted radiotherapy'

8. 4 OR 5 OR 6 OR 7

9. 'myasthenia gravis'/exp OR 'myasthenia gravis'

10. 3 AND 8

11. 10 AND 9

12. 10 NOT 9

13. 11 OR 12

14. Limits: Humans, English language

The Cochrane Library

#1 MeSH descriptor: [Thymoma] explode all trees

#2 MeSH descriptor: [Thymic Neoplasms] explode all trees

#3 thymoma*:ti,ab,kw

#4 thymic carcinoma*:ti,ab,kw

#5 (#1 OR #2 OR #3 OR #4)

#6 MeSH descriptor: [Radiotherapy] explode all trees

#7 MeSH descriptor: [Postoperative Care] explode all trees

#8 postoperative radiotherapy:ti,ab,kw

#9 radiation treatment*:ti,ab,kw

#10 targeted radiotherapy:ti,ab,kw

#11 (#6 OR #7 OR #8 OR #9 OR #10)

#12 MeSH descriptor: [Myasthenia Gravis] explode all trees

#13 myasthenia gravis:ti,ab,kw

#14 (#12 OR #13)

#15 (#5 AND #11)

#16 (#15 AND #14)

#17 (#15 NOT #14)

#18 (#16 OR #17)

Web of Science

(TS=(thymoma OR "thymic carcinoma*") AND

TS=("postoperative radiotherapy" OR radiotherapy OR "radiation treatment*" OR "targeted radiotherapy")) AND

(TS=("myasthenia gravis") OR NOT TS=("myasthenia gravis"))

Timespan: All years up to August 2024

Databases: Science Citation Index Expanded (SCI-EXPANDED), Social Sciences Citation Index (SSCI)

Document Types: Article, Review

Languages: English

Wanfang Database

#1 Topic: (Thymoma OR Thymic Neoplasm OR Thymic Carcinoma)

#2 Topic: (Postoperative Radiotherapy OR Radiotherapy OR Radiation Treatment OR Targeted Radiotherapy)

#3 Topic: (Myasthenia Gravis)

#4 #1 AND #2

#5 #4 AND #3

#6 #4 NOT #3

#7 #5 OR #6

#8 Time range: From inception to August 2024

#9 Document types: Journal articles, Dissertations, Conference papers

#10 Language: English

VIP Database

#1 (Keyword=Thymoma OR Keyword=Thymic Neoplasm OR Keyword=Thymic Carcinoma)

#2 (Keyword=Postoperative Radiotherapy OR Keyword=Radiotherapy OR Keyword=Radiation Treatment OR Keyword=Targeted Radiotherapy)

#3 (Keyword=Myasthenia Gravis)

#4 #1 AND #2

#5 #4 AND #3

#6 #4 NOT #3

#7 #5 OR #6

#8 Publication time: All years up to August 2024

#9 Document types: Journal articles, Dissertations

China National Knowledge Infrastructure (CNKI)

#1 Topic: Thymoma OR Thymic Neoplasm OR Thymic Carcinoma

#2 Topic: Postoperative Radiotherapy OR Radiotherapy OR Radiation Treatment OR Targeted Radiotherapy

#3 Topic: Myasthenia Gravis

#4 #1 AND #2

#5 #4 AND #3

#6 #4 NOT #3

#7 #5 OR #6

#8 Time range: 1979-August 2024

#9 Source types: Journals, Dissertations, Conference papers

#10 Language: English
