# Supplementary material for: The effectiveness of adjuvant radiotherapy after thymoma resection: a systematic review and meta-analysis
Source: World J Surg Oncol. 2025 Dec 10;24:36. doi: 10.1186/s12957-025-04127-z (PMC12801928; doi:10.1186/s12957-025-04127-z)
Supplement: Supplementary file 2 — Supplementary Material 2. [file 12957_2025_4127_MOESM2_ESM.docx]

**Table 1 Literature inclusion process**

Databases were searched（n=651）

Literature obtained by other means（n=0）

Removal of duplicate contributions（n=187）

Literature was excluded from the primary screening（n=360）

The number of articles that evaluated the full text（n=104）

The type of article does not match（n=16）

The required outcome measures were not available（n=26）

The quality of the literature was low（n=39）

Full-text re-screening of literature（n=23）

Literature included in the meta-analysis（n=23）

PubMed（n=411）、EMbase（n=108）The Cochrane Library（n=4）、Web of Science（n=42）、CNKI（n=58）、Wanfang Data（n=18）、VIP（n=10）
