# Supplementary figures and images for: The effectiveness of adjuvant radiotherapy after thymoma resection: a systematic review and meta-analysis
Source: World J Surg Oncol. 2025 Dec 10;24:36. doi: 10.1186/s12957-025-04127-z (PMC12801928; doi:10.1186/s12957-025-04127-z)

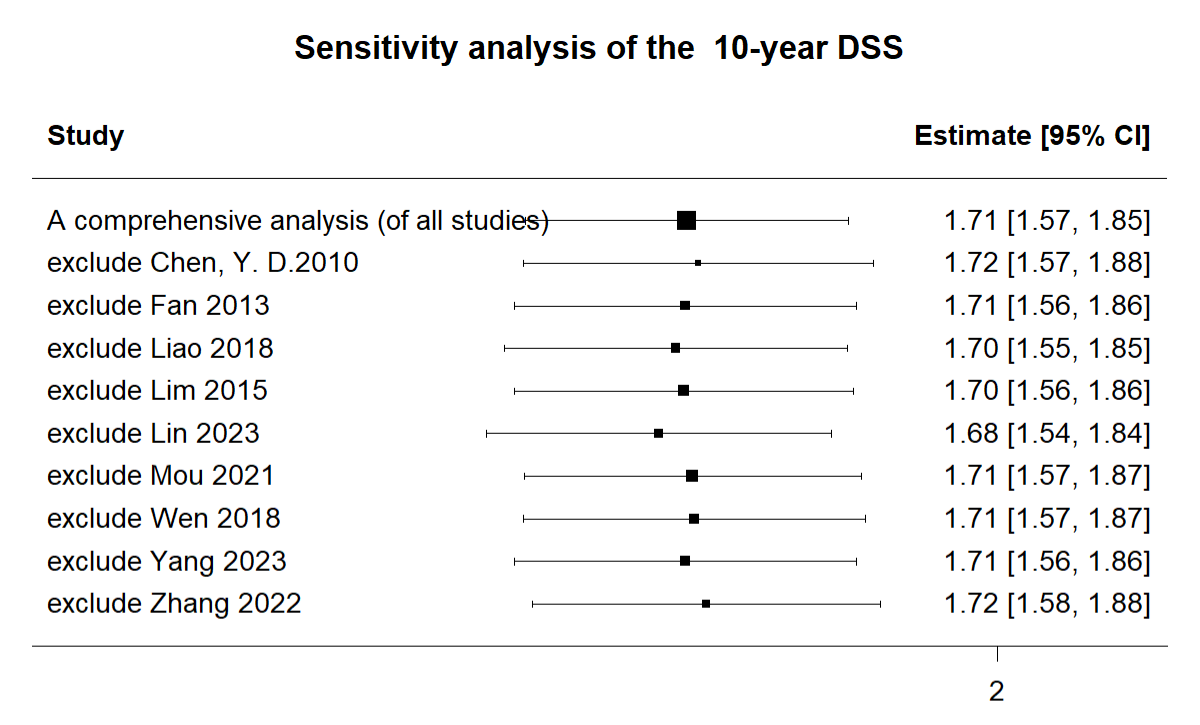

Supplement: Supplementary file 23 — Supplementary Material 23. [file 12957_2025_4127_MOESM23_ESM.png]

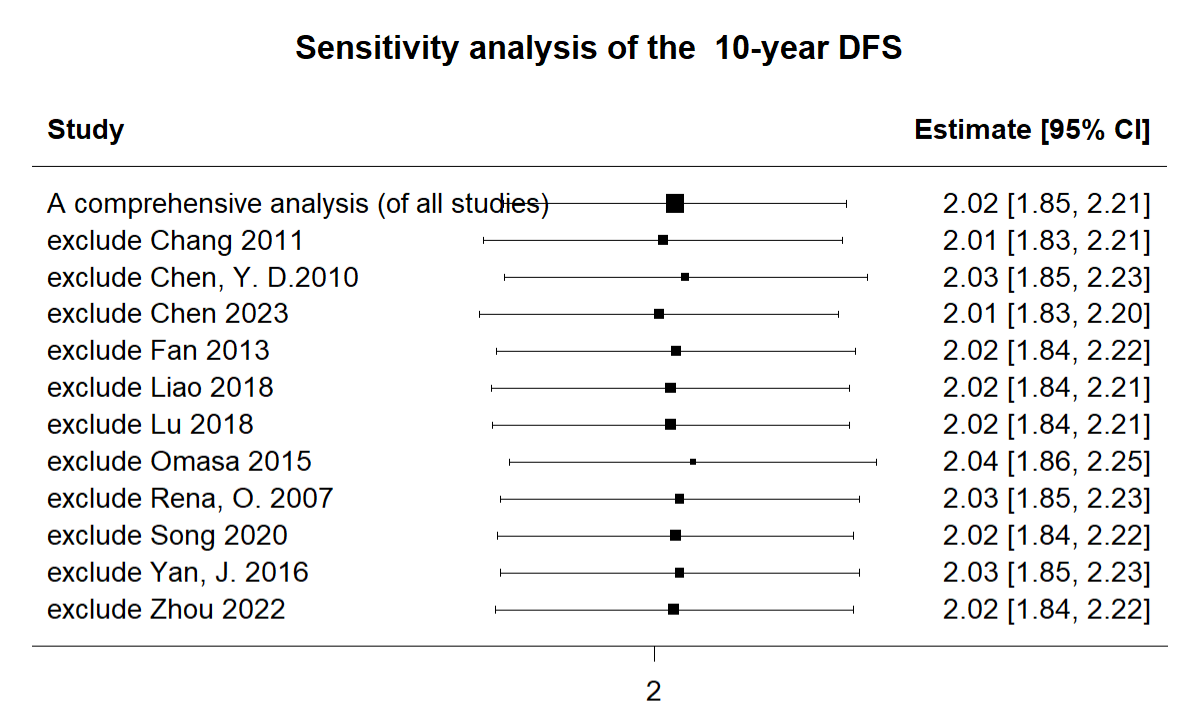

Supplement: Supplementary file 24 — Supplementary Material 24. [file 12957_2025_4127_MOESM24_ESM.png]

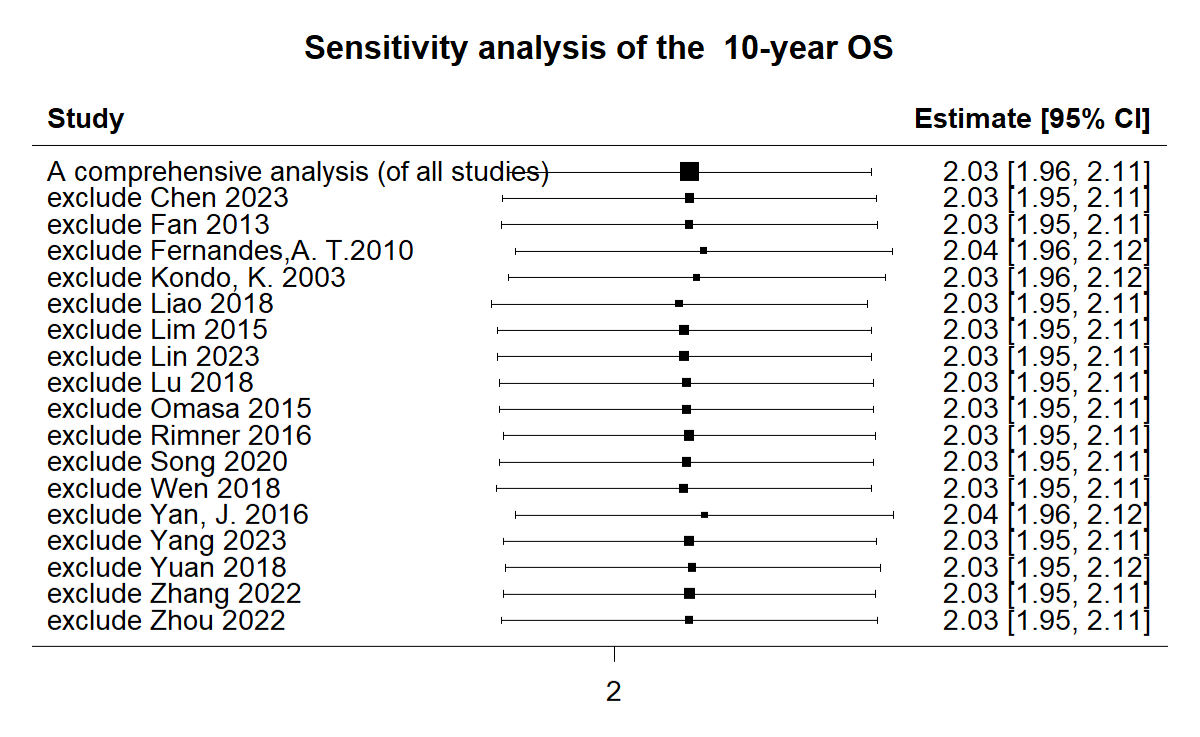

Supplement: Supplementary file 25 — Supplementary Material 25. [file 12957_2025_4127_MOESM25_ESM.png]
