# Supplementary material for: The effectiveness of adjuvant radiotherapy after thymoma resection: a systematic review and meta-analysis
Source: World J Surg Oncol. 2025 Dec 10;24:36. doi: 10.1186/s12957-025-04127-z (PMC12801928; doi:10.1186/s12957-025-04127-z)
Supplement: Supplementary file 27 — Supplementary Material 27. [file 12957_2025_4127_MOESM27_ESM.docx]

**Identification of studies via databases and registers**

Records removed *before screening*:

Duplicate records removed (n =187 )

Records marked as ineligible by automation tools (n = 0)

Records removed for other reasons (n = 0)

Records identified from*:

Databases (n = 651)

Registers (n =0 )

**Identification**

Records screened

(n =104 )

Records excluded**

(n = 360)

Reports sought for retrieval

(n = 104)

Reports not retrieved

(n =0 )

**Screening**

Reports assessed for eligibility

(n =104 )

Reports excluded:

The type of article dose not match (n =16 )

The quality of the literature was low(n =39 )

The required outcome measures were not available (n =26 )

etc.

Studies included in review

(n = 23)

Reports of included studies

(n =23 )

**Included**

*Consider, if feasible to do so, reporting the number of records identified from each database or register searched (rather than the total number across all databases/registers).

**If automation tools were used, indicate how many records were excluded by a human and how many were excluded by automation tools.

Source: Page MJ, et al. BMJ 2021;372:n71. doi: 10.1136/bmj.n71.

This work is licensed under CC BY 4.0. To view a copy of this license, visit <https://creativecommons.org/licenses/by/4.0/>
